# Triclinic polymorph of dibenzotetra­thia­fulvalene

**DOI:** 10.1107/S1600536809030013

**Published:** 2009-08-08

**Authors:** Masashi Mamada, Yoshiro Yamashita

**Affiliations:** a Department of Electronic Chemistry, Interdisciplinary Graduate School of Science and Engineering, Tokyo Institute of Technology, Nagatsuta, Midori-ku, Yokohama 226-8502, Japan

## Abstract

Crystals of the title compound (DBTTF), C_14_H_8_S_4_, feature a triclinic polymorph different from two known monoclinic polymorphs. In this form, there are two independent centrosymmetric half-mol­ecules in the asymmetric unit. Although the mol­ecular orientations are relatively similar to one of monoclinic polymorphs, the packing motif is different.

## Related literature

For the synthesis, see: Nakayama *et al.* (1976 ▶). For the monoclinic polymorphs of DBTTF, see: Emge *et al.* (1982 ▶); Brillante *et al.* (2008 ▶). For the electronic properties of DBTTF, see: Jigami *et al.* (1998 ▶). For the characteristics of field-effect transistors based on DBTTF, see, for example: Mas-Torrent *et al.* (2005 ▶); Shibata *et al.* (2008 ▶). For related structures, see: Mas-Torrent *et al.* (2004 ▶); Naraso *et al.* (2006 ▶).
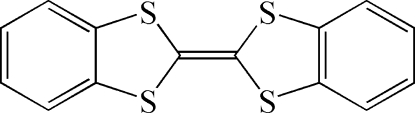


## Experimental

### 

#### Crystal data

C_14_H_8_S_4_
*M* *_r_* = 304.46
Triclinic, 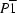

*a* = 8.6562 (4) Å
*b* = 9.4144 (5) Å
*c* = 9.5144 (4) Å
α = 74.0424 (15)°
β = 63.6158 (13)°
γ = 65.5653 (14)°
*V* = 628.43 (5) Å^3^
*Z* = 2
Mo *K*α radiation
μ = 0.73 mm^−1^
*T* = 93 K
0.35 × 0.25 × 0.15 mm

#### Data collection

Rigaku R-AXIS RAPID diffractometer
Absorption correction: multi-scan (*ABSCOR*; Higashi, 1995 ▶) *T*_min_ = 0.663, *T*_max_ = 0.896
6216 measured reflections
2871 independent reflections
2193 reflections with *F* ^2^ > 2σ(*F* ^2^)
*R*_int_ = 0.028

#### Refinement

*R*[*F* ^2^ > 2σ(*F* ^2^)] = 0.037
*wR*(*F* ^2^) = 0.110
*S* = 1.12
2871 reflections
164 parameters
All H-atom parameters refined
Δρ_max_ = 0.56 e Å^−3^
Δρ_min_ = −0.46 e Å^−3^

### 

Data collection: *PROCESS-AUTO* (Rigaku, 1998 ▶); cell refinement: *PROCESS-AUTO*; data reduction: *CrystalStructure* (Rigaku/MSC, 2007 ▶); program(s) used to solve structure: *SHELXS97* (Sheldrick, 2008 ▶); program(s) used to refine structure: *SHELXL97* (Sheldrick, 2008 ▶); molecular graphics: *ORTEP-3* (Farrugia, 1997 ▶); software used to prepare material for publication: *CrystalStructure*.

## Supplementary Material

Crystal structure: contains datablocks global, I. DOI: 10.1107/S1600536809030013/bt5019sup1.cif

Structure factors: contains datablocks I. DOI: 10.1107/S1600536809030013/bt5019Isup2.hkl

Additional supplementary materials:  crystallographic information; 3D view; checkCIF report

## Figures and Tables

**Fig. 1.**
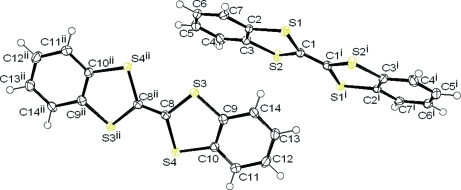
The molecular structure of (I), showing 50% probability displacement ellipsoids. [symmetry codes: (i) 1 - x, 1 - y, 1 - z, (ii) -x, -y, 1 - z.]

**Fig. 2.**
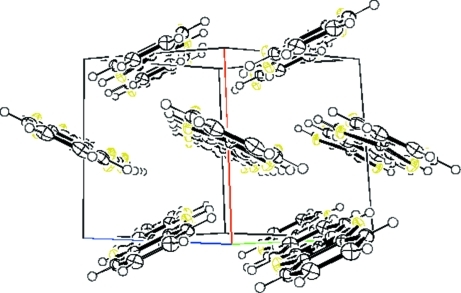
A packing diagram for (I) viewed along the molecular long axis.

## supplementary crystallographic information

### Comment

TTF and its derivatives have been well known as the most dominant electron donors (Jigami *et al.*, 1998). Recently, some TTF derivatives have been used as organic semiconductors for organic field-effect transistors (OFETs) (Mas-Torrent *et al.*, 2004; Naraso *et al.*, 2006), which show high hole mobilities in both the single crystals and thin films. The title compound, dibenzotetrathiafulvalene (I), has also exhibited high mobility (Mas-Torrent *et al.*, 2005; Shibata *et al.*, 2008). Since the strong intermolecular interactions between the molecules are necessary for carrier transportation in organic conductors and semiconductors, the investigation of crystal structures are very important. The crystal structure of compound (I) has been reported as two monoclinic polymorphs, which are space group *P2_1_/c* for α phase (Emge *et al.*, 1982) and *Cc* for β phase (Brillante *et al.*, 2008). These crystals were grown from solution and the existence of other two polymorphs has been found by means of lattice phonon confocal Raman microscopy and XRD. Herein, we report the single-crystal structure of compound (I) neither the α nor β phase.

The single-crystal of (I) which has been grown by vapour transport contains two crystallographically independent molecules with an inversion center. The molecules have chair-like structures which are slightly distorted from the molecular plane. The maximum deviations from the least-squares plane are 0.206 and 0.222 Å, and the average deviations are 0.085 and 0.111 Å. The molecular geometries in this phase are different from that in the α phase (average deviation: 0.037 Å) and similar to that in the β phase (maximum deviation: 0.235 Å, average deviation: 0.085 Å). The packing structure in this phase is a herringbone type with a tilt angle of 51.11° (Fig. 2), which is also similar to the β phase. However, the long axis is observed to be sliding and the number of intermolecular short contacts between the molecules is increased and the contact distances are shorter compared with the α and the β phase.

### Experimental

To a stirred solution of 1,3-benzodithiolylium tetrafluoroborate (0.83 g, 3.5 mmol) in dichloromethane (30 ml) was added dropwise 1,8-diazabicyclo[5.4.0]undec-7-ene (1 ml) at room temperature. After being stirred for 4 h, the resulting precipitate was filtered, washed with dichloromethane and dried to give 0.30 g (58%) of the title compound. Yellow crystals of (I) suitable for X-ray analysis were obtained from slow vacuum sublimation in a gradient-temperature horizontal glass tube. A temperature of the source material was maintained at 480 K.

### Refinement

H atoms were placed in calculated positions and refined in the riding model, with C—H = 0.95 Å and *U_iso_*(H) = 1.2*U_eq_*(C) for CH groups.

### Crystal data

**Table:**

C_14_H_8_S_4_	*Z* = 2
*M**_r_* = 304.46	*F*(000) = 312.00
Triclinic, *P*1	*D*_x_ = 1.609 Mg m^−^^3^
Hall symbol: -P 1	Mo *K*α radiation, λ = 0.71075 Å
*a* = 8.6562 (4) Å	Cell parameters from 4982 reflections
*b* = 9.4144 (5) Å	θ = 3.0–27.5°
*c* = 9.5144 (4) Å	µ = 0.73 mm^−^^1^
α = 74.0424 (15)°	*T* = 93 K
β = 63.6158 (13)°	Platelet, yellow
γ = 65.5653 (14)°	0.35 × 0.25 × 0.15 mm
*V* = 628.43 (5) Å^3^	

### Data collection

**Table:**

Rigaku R-AXIS RAPID diffractometer	2193 reflections with *F*^2^ > 2σ(*F*^2^)
Detector resolution: 10.00 pixels mm^-1^	*R*_int_ = 0.028
ω scans	θ_max_ = 27.5°
Absorption correction: multi-scan (*ABSCOR*; Higashi, 1995)	*h* = −11→11
*T*_min_ = 0.663, *T*_max_ = 0.896	*k* = −12→12
6216 measured reflections	*l* = −12→11
2871 independent reflections	

### Refinement

**Table:**

Refinement on *F*^2^	All H-atom parameters refined
*R*[*F*^2^ > 2σ(*F*^2^)] = 0.037	*w* = 1/[σ^2^(*F*_o_^2^) + (0.0363*P*)^2^ + 1.1654*P*] where *P* = (*F*_o_^2^ + 2*F*_c_^2^)/3
*wR*(*F*^2^) = 0.110	(Δ/σ)_max_ < 0.001
*S* = 1.12	Δρ_max_ = 0.56 e Å^−^^3^
2871 reflections	Δρ_min_ = −0.46 e Å^−^^3^
164 parameters	

### Special details

**Table:**

Geometry. ENTER SPECIAL DETAILS OF THE MOLECULAR GEOMETRY
Refinement. Refinement was performed using all reflections. The weighted *R*-factor (*wR*) and goodness of fit (*S*) are based on *F*^2^. *R*-factor (gt) are based on *F*. The threshold expression of *F*^2^ > 2.0 σ(*F*^2^) is used only for calculating *R*-factor (gt).

### Fractional atomic coordinates and isotropic or equivalent isotropic displacement parameters (Å^2^)

**Table:**

	*x*	*y*	*z*	*U*_iso_*/*U*_eq_	
S(1)	0.43963 (10)	0.53797 (9)	0.28891 (8)	0.01843 (17)	
S(2)	0.60882 (10)	0.24998 (8)	0.46970 (9)	0.02064 (17)	
S(3)	0.07081 (10)	0.20979 (9)	0.46015 (9)	0.01946 (17)	
S(4)	−0.11044 (10)	0.02886 (9)	0.75183 (8)	0.01913 (17)	
C(1)	0.5095 (3)	0.4558 (3)	0.4497 (3)	0.0156 (5)	
C(2)	0.4701 (3)	0.3575 (3)	0.2425 (3)	0.0172 (5)	
C(3)	0.5503 (3)	0.2223 (3)	0.3267 (3)	0.0193 (5)	
C(4)	0.5830 (4)	0.0743 (3)	0.2954 (4)	0.0241 (6)	
C(5)	0.5339 (4)	0.0630 (4)	0.1786 (4)	0.0297 (7)	
C(6)	0.4545 (4)	0.1975 (4)	0.0947 (3)	0.0291 (7)	
C(7)	0.4203 (4)	0.3460 (4)	0.1263 (3)	0.0232 (6)	
C(8)	−0.0073 (3)	0.0491 (3)	0.5443 (3)	0.0161 (5)	
C(9)	0.0398 (3)	0.2558 (3)	0.6390 (3)	0.0181 (5)	
C(10)	−0.0446 (3)	0.1709 (3)	0.7768 (3)	0.0173 (5)	
C(11)	−0.0710 (4)	0.1986 (3)	0.9235 (3)	0.0222 (6)	
C(12)	−0.0092 (4)	0.3109 (4)	0.9306 (4)	0.0270 (7)	
C(13)	0.0748 (4)	0.3954 (3)	0.7934 (4)	0.0265 (6)	
C(14)	0.0984 (4)	0.3703 (3)	0.6477 (3)	0.0211 (6)	
H(1)	0.6380	−0.0176	0.3526	0.029*	
H(2)	0.5550	−0.0373	0.1565	0.036*	
H(3)	0.4231	0.1883	0.0147	0.035*	
H(4)	0.3641	0.4378	0.0697	0.028*	
H(5)	−0.1302	0.1420	1.0174	0.027*	
H(6)	−0.0247	0.3296	1.0299	0.032*	
H(7)	0.1166	0.4713	0.7998	0.032*	
H(8)	0.1536	0.4299	0.5544	0.025*	

### Atomic displacement parameters (Å^2^)

**Table:**

	*U*^11^	*U*^22^	*U*^33^	*U*^12^	*U*^13^	*U*^23^
S(1)	0.0239 (3)	0.0176 (3)	0.0167 (3)	−0.0073 (2)	−0.0113 (2)	0.0003 (2)
S(2)	0.0255 (3)	0.0145 (3)	0.0260 (3)	−0.0040 (2)	−0.0158 (3)	−0.0021 (3)
S(3)	0.0250 (3)	0.0186 (3)	0.0157 (3)	−0.0117 (3)	−0.0055 (2)	0.0000 (2)
S(4)	0.0247 (3)	0.0210 (3)	0.0144 (3)	−0.0139 (3)	−0.0053 (2)	0.0002 (2)
C(1)	0.0180 (13)	0.0138 (12)	0.0156 (12)	−0.0057 (11)	−0.0079 (10)	0.0008 (11)
C(2)	0.0152 (12)	0.0240 (14)	0.0145 (12)	−0.0101 (11)	−0.0017 (10)	−0.0065 (11)
C(3)	0.0177 (13)	0.0227 (14)	0.0187 (13)	−0.0077 (11)	−0.0043 (11)	−0.0069 (12)
C(4)	0.0185 (14)	0.0227 (15)	0.0308 (16)	−0.0098 (12)	−0.0042 (12)	−0.0068 (13)
C(5)	0.0276 (16)	0.0363 (18)	0.0293 (16)	−0.0187 (15)	−0.0003 (13)	−0.0150 (15)
C(6)	0.0311 (16)	0.048 (2)	0.0188 (14)	−0.0263 (16)	−0.0015 (13)	−0.0121 (15)
C(7)	0.0245 (15)	0.0366 (18)	0.0138 (13)	−0.0180 (14)	−0.0046 (11)	−0.0031 (13)
C(8)	0.0163 (12)	0.0164 (13)	0.0155 (12)	−0.0058 (11)	−0.0077 (10)	0.0017 (11)
C(9)	0.0156 (12)	0.0167 (13)	0.0223 (14)	−0.0043 (11)	−0.0079 (11)	−0.0032 (12)
C(10)	0.0164 (12)	0.0151 (13)	0.0192 (13)	−0.0036 (10)	−0.0058 (11)	−0.0046 (11)
C(11)	0.0209 (14)	0.0243 (15)	0.0194 (14)	−0.0075 (12)	−0.0046 (12)	−0.0049 (13)
C(12)	0.0293 (16)	0.0291 (17)	0.0252 (16)	−0.0077 (14)	−0.0083 (13)	−0.0135 (14)
C(13)	0.0271 (15)	0.0256 (16)	0.0305 (16)	−0.0112 (13)	−0.0073 (13)	−0.0109 (14)
C(14)	0.0193 (13)	0.0197 (14)	0.0253 (15)	−0.0101 (12)	−0.0061 (12)	−0.0024 (12)

### Geometric parameters (Å, °)

**Table:**

S(1)—C(1)	1.758 (3)	C(8)—C(8)^ii^	1.350 (5)
S(1)—C(2)	1.757 (3)	C(9)—C(10)	1.395 (3)
S(2)—C(1)	1.759 (2)	C(9)—C(14)	1.401 (5)
S(2)—C(3)	1.759 (4)	C(10)—C(11)	1.391 (5)
S(3)—C(8)	1.756 (3)	C(11)—C(12)	1.395 (6)
S(3)—C(9)	1.748 (3)	C(12)—C(13)	1.389 (4)
S(4)—C(8)	1.760 (2)	C(13)—C(14)	1.382 (5)
S(4)—C(10)	1.759 (4)	C(4)—H(1)	0.950
C(1)—C(1)^i^	1.349 (5)	C(5)—H(2)	0.950
C(2)—C(3)	1.395 (4)	C(6)—H(3)	0.950
C(2)—C(7)	1.396 (5)	C(7)—H(4)	0.950
C(3)—C(4)	1.391 (5)	C(11)—H(5)	0.950
C(4)—C(5)	1.396 (6)	C(12)—H(6)	0.950
C(5)—C(6)	1.388 (4)	C(13)—H(7)	0.950
C(6)—C(7)	1.393 (6)	C(14)—H(8)	0.950
			
S(1)···C(7)^iii^	3.568 (3)	H(1)···C(9)^vi^	2.782
S(1)···C(10)^iv^	3.578 (2)	H(1)···C(10)^vi^	2.985
S(2)···C(8)^v^	3.359 (3)	H(1)···C(11)^vi^	3.504
S(2)···C(8)^vi^	3.407 (2)	H(1)···C(14)^vi^	3.156
S(3)···C(6)	3.574 (2)	H(1)···H(1)^vi^	2.754
C(1)···C(14)	3.527 (4)	H(1)···H(5)^x^	3.260
C(4)···C(4)^vi^	3.589 (4)	H(1)···H(8)^vi^	3.583
C(4)···C(10)^vi^	3.545 (3)	H(2)···S(2)^vi^	3.552
C(6)···S(3)	3.574 (2)	H(2)···S(4)^ii^	3.518
C(7)···S(1)^iii^	3.568 (3)	H(2)···C(6)^xi^	3.202
C(7)···C(7)^iii^	3.552 (5)	H(2)···C(12)^vi^	3.561
C(8)···S(2)^vii^	3.359 (3)	H(2)···H(2)^xi^	3.329
C(8)···S(2)^vi^	3.407 (2)	H(2)···H(3)^xi^	2.341
C(10)···S(1)^iv^	3.578 (2)	H(2)···H(5)^x^	3.376
C(10)···C(4)^vi^	3.545 (3)	H(3)···S(1)^iii^	3.433
C(11)···C(11)^viii^	3.523 (4)	H(3)···S(4)^x^	3.590
C(14)···C(1)	3.527 (4)	H(3)···C(5)^xi^	3.189
S(1)···H(3)^iii^	3.433	H(3)···H(2)^xi^	2.341
S(1)···H(4)^iii^	3.053	H(3)···H(3)^xi^	3.275
S(1)···H(7)^i^	3.515	H(3)···H(6)^xiii^	3.486
S(1)···H(8)	2.942	H(4)···S(1)^iii^	3.053
S(2)···H(1)^vi^	3.291	H(4)···C(2)^iii^	3.154
S(2)···H(2)^vi^	3.552	H(4)···C(7)^iii^	3.010
S(2)···H(8)	3.384	H(4)···C(11)^iv^	3.309
S(3)···H(1)^vi^	3.354	H(4)···C(12)^iv^	3.004
S(3)···H(8)^iv^	3.135	H(4)···C(13)^iv^	3.223
S(4)···H(2)^ii^	3.518	H(4)···H(4)^iii^	2.705
S(4)···H(3)^ix^	3.590	H(4)···H(6)^iv^	3.215
S(4)···H(5)^viii^	3.332	H(4)···H(7)^iv^	3.555
S(4)···H(6)^viii^	3.480	H(5)···S(4)^viii^	3.332
C(1)···H(7)	3.532	H(5)···C(2)^ix^	3.267
C(1)···H(7)^i^	3.271	H(5)···C(3)^ix^	3.024
C(1)···H(8)	2.881	H(5)···C(4)^ix^	2.848
C(1)···H(8)^i^	3.484	H(5)···C(5)^ix^	2.925
C(2)···H(4)^iii^	3.154	H(5)···C(6)^ix^	3.169
C(2)···H(5)^x^	3.267	H(5)···C(7)^ix^	3.351
C(2)···H(8)	3.019	H(5)···C(10)^viii^	3.252
C(3)···H(1)^vi^	3.259	H(5)···C(11)^viii^	2.982
C(3)···H(5)^x^	3.024	H(5)···H(1)^ix^	3.260
C(3)···H(8)	3.235	H(5)···H(2)^ix^	3.376
C(4)···H(1)^vi^	3.055	H(5)···H(5)^viii^	2.679
C(4)···H(5)^x^	2.848	H(6)···S(4)^viii^	3.480
C(5)···H(3)^xi^	3.189	H(6)···C(13)^xii^	3.245
C(5)···H(5)^x^	2.925	H(6)···H(3)^xiv^	3.486
C(6)···H(2)^xi^	3.202	H(6)···H(4)^iv^	3.215
C(6)···H(5)^x^	3.169	H(6)···H(6)^xii^	3.273
C(7)···H(4)^iii^	3.010	H(6)···H(7)^xii^	2.461
C(7)···H(5)^x^	3.351	H(7)···S(1)^i^	3.515
C(9)···H(1)^vi^	2.782	H(7)···C(1)	3.532
C(9)···H(8)^iv^	3.254	H(7)···C(1)^i^	3.271
C(10)···H(1)^vi^	2.985	H(7)···C(12)^xii^	3.293
C(10)···H(5)^viii^	3.252	H(7)···H(4)^iv^	3.555
C(11)···H(1)^vi^	3.504	H(7)···H(6)^xii^	2.461
C(11)···H(4)^iv^	3.309	H(7)···H(7)^xii^	3.503
C(11)···H(5)^viii^	2.982	H(8)···S(1)	2.942
C(12)···H(2)^vi^	3.561	H(8)···S(2)	3.384
C(12)···H(4)^iv^	3.004	H(8)···S(3)^iv^	3.135
C(12)···H(7)^xii^	3.293	H(8)···C(1)	2.881
C(13)···H(4)^iv^	3.223	H(8)···C(1)^i^	3.484
C(13)···H(6)^xii^	3.245	H(8)···C(2)	3.019
C(14)···H(1)^vi^	3.156	H(8)···C(3)	3.235
C(14)···H(8)^iv^	3.215	H(8)···C(9)^iv^	3.254
H(1)···S(2)^vi^	3.291	H(8)···C(14)^iv^	3.215
H(1)···S(3)^vi^	3.354	H(8)···H(1)^vi^	3.583
H(1)···C(3)^vi^	3.259	H(8)···H(8)^iv^	2.946
H(1)···C(4)^vi^	3.055		
			
C(1)—S(1)—C(2)	95.02 (15)	S(4)—C(10)—C(9)	116.1 (2)
C(1)—S(2)—C(3)	95.06 (16)	S(4)—C(10)—C(11)	123.3 (2)
C(8)—S(3)—C(9)	95.27 (15)	C(9)—C(10)—C(11)	120.5 (3)
C(8)—S(4)—C(10)	95.27 (16)	C(10)—C(11)—C(12)	119.0 (2)
S(1)—C(1)—S(2)	115.5 (2)	C(11)—C(12)—C(13)	120.5 (3)
S(1)—C(1)—C(1)^i^	122.5 (2)	C(12)—C(13)—C(14)	120.7 (4)
S(2)—C(1)—C(1)^i^	122.0 (2)	C(9)—C(14)—C(13)	119.3 (2)
S(1)—C(2)—C(3)	116.8 (3)	C(3)—C(4)—H(1)	120.5
S(1)—C(2)—C(7)	122.9 (2)	C(5)—C(4)—H(1)	120.5
C(3)—C(2)—C(7)	120.3 (3)	C(4)—C(5)—H(2)	119.8
S(2)—C(3)—C(2)	116.6 (2)	C(6)—C(5)—H(2)	119.8
S(2)—C(3)—C(4)	122.8 (2)	C(5)—C(6)—H(3)	119.6
C(2)—C(3)—C(4)	120.6 (3)	C(7)—C(6)—H(3)	119.5
C(3)—C(4)—C(5)	119.0 (3)	C(2)—C(7)—H(4)	120.6
C(4)—C(5)—C(6)	120.3 (4)	C(6)—C(7)—H(4)	120.6
C(5)—C(6)—C(7)	120.9 (4)	C(10)—C(11)—H(5)	120.5
C(2)—C(7)—C(6)	118.8 (3)	C(12)—C(11)—H(5)	120.5
S(3)—C(8)—S(4)	115.1 (2)	C(11)—C(12)—H(6)	119.8
S(3)—C(8)—C(8)^ii^	122.2 (2)	C(13)—C(12)—H(6)	119.8
S(4)—C(8)—C(8)^ii^	122.6 (2)	C(12)—C(13)—H(7)	119.6
S(3)—C(9)—C(10)	117.1 (3)	C(14)—C(13)—H(7)	119.7
S(3)—C(9)—C(14)	122.9 (2)	C(9)—C(14)—H(8)	120.4
C(10)—C(9)—C(14)	120.0 (3)	C(13)—C(14)—H(8)	120.4
			
C(1)—S(1)—C(2)—C(3)	−6.4 (2)	C(3)—C(2)—C(7)—C(6)	−0.9 (4)
C(1)—S(1)—C(2)—C(7)	174.5 (2)	C(7)—C(2)—C(3)—S(2)	179.7 (2)
C(2)—S(1)—C(1)—S(2)	10.26 (18)	C(7)—C(2)—C(3)—C(4)	0.5 (4)
C(2)—S(1)—C(1)—C(1)^i^	−170.5 (2)	S(2)—C(3)—C(4)—C(5)	−179.3 (2)
C(1)—S(2)—C(3)—C(2)	5.7 (2)	C(2)—C(3)—C(4)—C(5)	−0.2 (4)
C(1)—S(2)—C(3)—C(4)	−175.2 (2)	C(3)—C(4)—C(5)—C(6)	0.3 (4)
C(3)—S(2)—C(1)—S(1)	−10.05 (18)	C(4)—C(5)—C(6)—C(7)	−0.8 (4)
C(3)—S(2)—C(1)—C(1)^i^	170.7 (2)	C(5)—C(6)—C(7)—C(2)	1.0 (4)
C(8)—S(3)—C(9)—C(10)	−5.7 (2)	S(3)—C(8)—C(8)^ii^—S(4)^ii^	1.6 (3)
C(8)—S(3)—C(9)—C(14)	173.1 (2)	S(4)—C(8)—C(8)^ii^—S(3)^ii^	−1.6 (3)
C(9)—S(3)—C(8)—S(4)	10.05 (18)	S(3)—C(9)—C(10)—S(4)	−0.5 (2)
C(9)—S(3)—C(8)—C(8)^ii^	−171.4 (2)	S(3)—C(9)—C(10)—C(11)	178.8 (2)
C(8)—S(4)—C(10)—C(9)	6.4 (2)	S(3)—C(9)—C(14)—C(13)	−177.6 (2)
C(8)—S(4)—C(10)—C(11)	−172.8 (2)	C(10)—C(9)—C(14)—C(13)	1.3 (4)
C(10)—S(4)—C(8)—S(3)	−10.26 (18)	C(14)—C(9)—C(10)—S(4)	−179.4 (2)
C(10)—S(4)—C(8)—C(8)^ii^	171.2 (2)	C(14)—C(9)—C(10)—C(11)	−0.1 (3)
S(1)—C(1)—C(1)^i^—S(2)^i^	0.8 (3)	S(4)—C(10)—C(11)—C(12)	178.2 (2)
S(2)—C(1)—C(1)^i^—S(1)^i^	−0.8 (3)	C(9)—C(10)—C(11)—C(12)	−1.0 (4)
S(1)—C(2)—C(3)—S(2)	0.5 (2)	C(10)—C(11)—C(12)—C(13)	1.0 (4)
S(1)—C(2)—C(3)—C(4)	−178.7 (2)	C(11)—C(12)—C(13)—C(14)	0.2 (3)
S(1)—C(2)—C(7)—C(6)	178.2 (2)	C(12)—C(13)—C(14)—C(9)	−1.3 (4)

Symmetry codes: (i) −*x*+1, −*y*+1, −*z*+1; (ii) −*x*, −*y*, −*z*+1; (iii) −*x*+1, −*y*+1, −*z*; (iv) −*x*, −*y*+1, −*z*+1; (v) *x*+1, *y*, *z*; (vi) −*x*+1, −*y*, −*z*+1; (vii) *x*−1, *y*, *z*; (viii) −*x*, −*y*, −*z*+2; (ix) *x*−1, *y*, *z*+1; (x) *x*+1, *y*, *z*−1; (xi) −*x*+1, −*y*, −*z*; (xii) −*x*, −*y*+1, −*z*+2; (xiii) *x*, *y*, *z*−1; (xiv) *x*, *y*, *z*+1.
